# Transient Transfection of the Zoonotic Parasite *Babesia microti*

**DOI:** 10.3390/pathogens9020108

**Published:** 2020-02-10

**Authors:** Mingming Liu, Shengwei Ji, Mohamed Abdo Rizk, Paul Franck Adjou Moumouni, Eloiza May Galon, Jixu Li, Yongchang Li, Weiqing Zheng, Byamukama Benedicto, Maria Agnes Tumwebaze, Masahito Asada, Xuenan Xuan

**Affiliations:** 1 National Research Center for Protozoan Diseases, Obihiro University of Agriculture and Veterinary Medicine, Obihiro, Hokkaido 080-8555, Japan; lmm_2010@hotmail.com (M.L.); jishengwei0903@hotmail.com (S.J.); mohamedabdorizk@gmail.com (M.A.R.); chakirou82@yahoo.fr (P.F.A.M.); eloizagalon@gmail.com (E.M.G.); JixuLi@hotmail.com (J.L.); YongchangLi8762017@outlook.com (Y.L.); zhengweiqing2001@gmail.com (W.Z.); benards.benedicto4@gmail.com (B.B.); tumwebazeaggie@gmail.com (M.A.T.); masada@obihiro.ac.jp (M.A.); 2 Department of Internal Medicine and Infectious Diseases, Faculty of Veterinary Medicine, Mansoura University, Mansoura 35516, Egypt; 3 Nanchang Center for Disease Control and Prevention, Nanchang 330038, China

**Keywords:** human babesiosis, *Babesia microti*, transient transfection

## Abstract

The development of genetic manipulation techniques has been reported in many protozoan parasites over the past few years. However, these techniques have not been established for *Babesia microti*. Here, we report the first successful transient transfection of *B. microti*. The plasmids containing the firefly luciferase reporter gene were transfected into *B. microti* by an AMAXA 4D Nucleofection system. Twenty-four-hour synchronization, the *5′-actin* promoter, program FA100, and 50 μg of plasmid DNA constituted the best conditions for the transient transfection of *B. microti*. This finding is the first step towards a stable transfection method for *B. microti*, which may contribute to a better understanding of the biology of the parasite.

## 1. Introduction

The protozoan parasite *Babesia microti* is the main agent of human babesiosis. This parasite invades and multiplies within red blood cells (RBCs) and infections vary greatly in their presentation depending on the age and immune competency of the host [1]. Severe symptoms are observed in neonates, or in older adults, possibly due to depressed cellular immunity and in the immunocompromised of any age, particularly splenectomized individuals [2]. In patients who were hospitalized with severe *B. microti* infection, death occurred in 10% of the cases. Mild disease caused by *B. microti* usually presents intermittent fever with general malaise and weakness [3]. 

The difficulties in identifying *B. microti* virulence factors and developing effective therapies for human babesiosis have been partly attributed to the lack of genetic manipulation tools [4]. The development of these techniques has been reported in bovine and canine *Babesia* parasites, including *B. bovis*, *B. ovata*, *B. bigemina*, *B. gibsoni* and *B. ovis* [5,6,7,8,9,10]. The application of transfection systems can lead to a better understanding of host–parasite interactions, the mechanisms underlying drug resistance and provides novel information for vaccine development and drug target discovery [11]. For example, transgenic *B. bovis* has been used to analyze gene functions and live vaccines against bovine babesiosis [12,13,14]. 

In this study, in order to identify the transfection condition for *B. microti*, the episome plasmids that contain promoters, firefly luciferase reporter, and terminator, were transfected into synchronized *B. microti* merozoites.

## 2. Results

**Figure 1:**
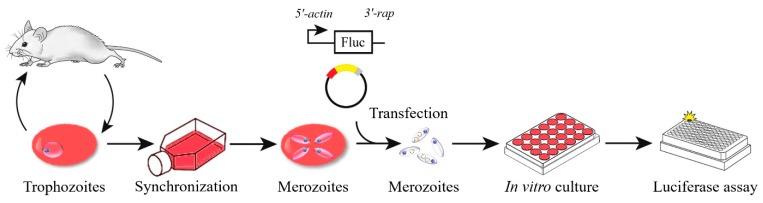
The schematic diagram of transient transfection of *B. microti*.

**Figure 2:**
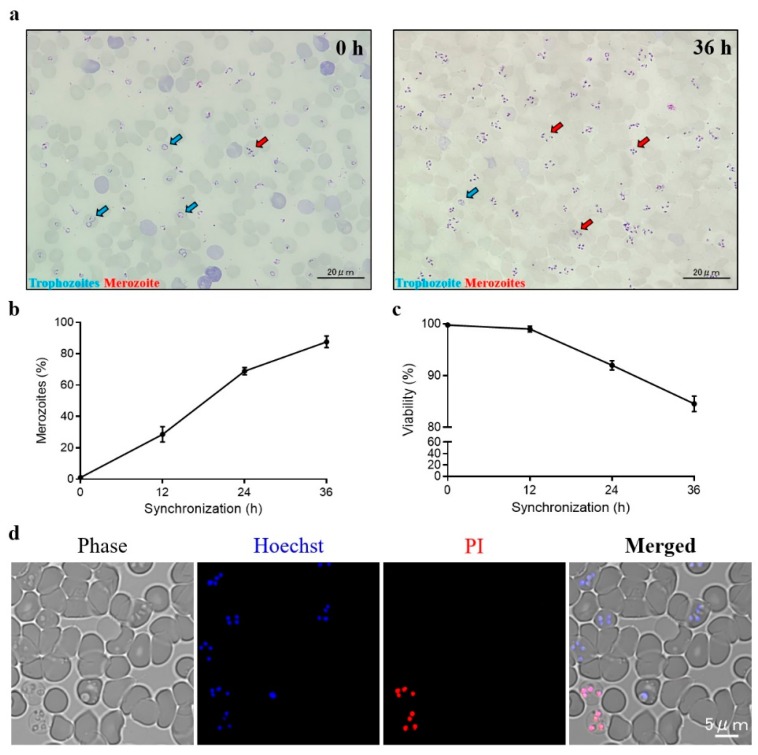
*B. microti* synchronization. (**a**) Unsynchronized (left) and 36-h (right) synchronized thin blood smears. (**b**) The percentages of merozoites were calculated by Giemsa staining. (**c**) Parasite viability were calculated by Hoechst 33342/Propidium iodide (PI) double staining. (**d**) Fluorescent microscope images of synchronized *B. microti*. Parasites were stained by Hoechst 33342/PI double staining at 36 h post-synchronization. Hoechst 33342 was used to stain the nuclei of both live and dead parasites. PI was used to stain the nuclei of dead parasites. The values are presented as a mean ± S.D. of three independent experiments.

**Figure 3:**
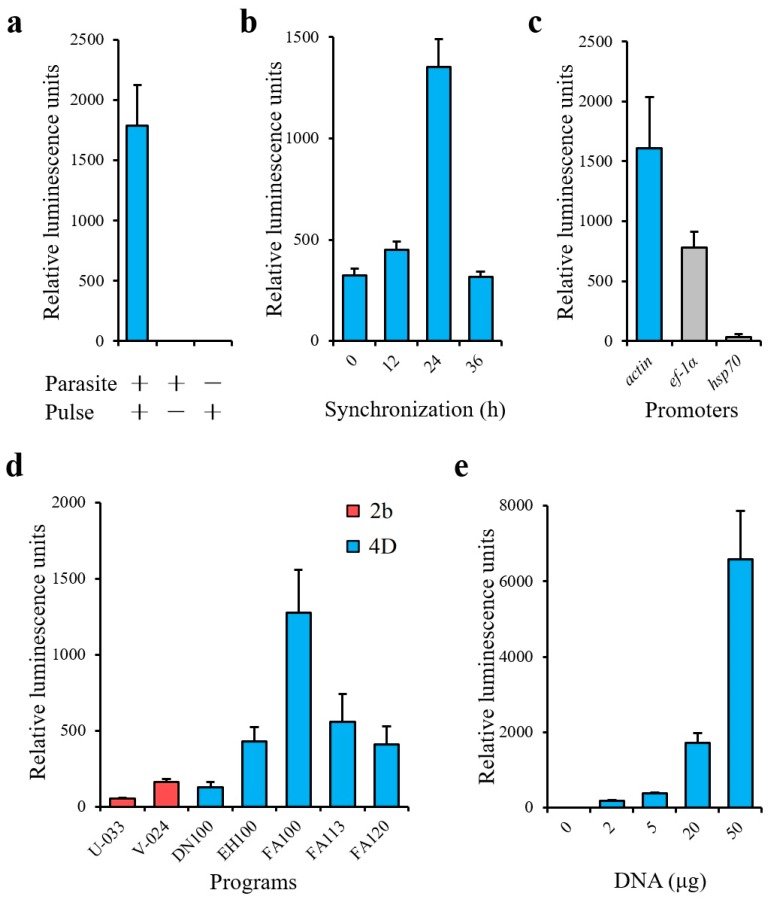
(**a**) Transient transfection of *B. microti*. (**b**) Transfection by various synchronized parasites. (**c**) Selection of promoters. *Actin* (*5′-actin*), *ef-1α* (*5′-ef-1α-subunit*) and *hsp70* (*5′-hsp70*). (**d**) Selection of programs. 2b (AMAXA Nucleofector 2b using Human T-cell buffer) and 4D (AMAXA 4D Nucleofector using an SF buffer). (**e**) Transfection by varying amounts of plasmid. Twenty-four-hour synchronized parasites were used in a, c, d, and e. *Actin* promoter was used in a, b, d, and e. Program FA100 was used in a, b, c, and e. Twenty micrograms of plasmid was used in a, b, c, and d. The values are presented as a mean ± S.D. of three independent experiments.

The schematic diagram of transient transfection described in this study is shown in Figure 1. During the time course of synchronization, parasite viability decreased while the percentage of merozoites significantly increased (Figure 2). Luminescence was shown to require the presence of a parasite and transfection pulse (Figure 3a). Twenty-four-hour synchronized parasites showed higher luciferase activity than the unsynchronized parasites or parasites with 12 and 36-h synchronization (Figure 3b). *5′-actin* was the best promoter out of the three candidates tested in this study (Figure 3c). Program FA100 showed higher luciferase activity than other programs (Figure 3d). Fifty micrograms of plasmid DNA was more efficient than 2, 5, and 20 μg (Figure 3e). The sequences of *B. microti 5′-actin*, *5′-ef-1α-subunit*, and *5′-hsp70* promoter were deposited into the GenBank database (accession numbers: MN891916-MN891918). The primers are listed in Table 1.

## 3. Discussion

In the United States, human babesiosis caused by *B. microti* is considered an emerging infectious disease. Aside from being transmitted by the hard tick, the majority of the cases are blood transfusion-transmitted babesiosis [16]. Increasing global interconnectivity raises the risk for *B. microti* infections spreading to other countries [17,18,19]. *B. microti* has the smallest nuclear genome among all apicomplexan parasites [20]. Genome-wide phylogenetic analyses indicate that *B. microti* defines a new clade in the phylum Apicomplexa. Additionally, the difference in both the copy number and organization of multigene families resulted in the phylogeny and life cycle of *B. microti* being significantly distant from those of other *Babesia* and *Theileria* parasites [21,22]. Therefore, the established transfection methods for other apicomplexan parasites may not be used for *B. microti*.

As expected, the common transfection system for bovine *Babesia* parasites, the AMAXA Nucleofector 2b transfection system (Lonza, Cologne, Germany), was not efficient (Figure 3d). The AMAXA 4D Nucleofector provides a more suitable transfection system for non-bovine *Babesia* parasites [15]. The transfection system of other parasites was established by this system [23]. However, the optimal transfection program is also different among those parasites, such as FA113 (*B. gibsoni*) and EH100 (*Cryptosporidium parvum*), and may not be applicable to *B. microti* transfection. Overall, the results presented in this study also indicate that 4D Nucleofection system was more efficient than previous systems for non-bovine *Babesia* parasites.

Trophozoites were considered to have low transfection efficiency in rodent malaria parasites [24]. A similar observation was noted in this study, as a high percentage of trophozoites (non-synchronization) did not provide good result for *B. microti* transfection (Figure 3b). Unfortunately, the purification of *B. microti* merozoites by using both a Nycodenz density-gradient and a Percoll density-gradient failed (data not shown). As observed for the purification of *Plasmodium berghei* schizonts, synchronization is needed to increase the percentage of merozoites for transfection [25]. However, over-synchronization can affect the efficiency of transfection (Figure 3b). Because *B. microti* can only be maintained in a short-term in vitro culture [26], regardless of the red blood cells’(RBCs’) condition or parasite viability, an over-synchronized parasite may be unsuitable for transfection (Figure 2c,d).

Compared to another human *Babesia* parasite *B. duncani* [27], a lack of an in vitro culture system for *B. microti* has severely impacted studies on the parasite, including the search for suitable diagnostic and blood screening markers. Especially for genetic manipulation study, the common drug selection systems used for *Babesia* parasites, such as human dihydrofolate reductase (hDHFR)/WR99210 and blasticidin-S deaminase (BSD)/bsd selection systems, cannot be used for *B. microti* [28]. Additionally, *B. microti* is not sensitive to pyrimethamine, which is the only drug for rodent malaria parasites selection in vivo [29,30]. Overall, stable transfection cannot be fully assessed based on the present study due to challenges such as a lack of a continuous in vitro culture system and unclear selection markers.

In summary, we present the first successful transient transfection of *B. microti*. This finding is the first step towards a stable transfection method for *B. microti*, which may contribute to a better understanding of the biology of the parasite and paves the way for the development of more effective molecular-based subunit vaccines and the discovery of novel drug targets for controlling human babesiosis. However, further efforts should be put into establishing a continuous in vitro culture system or finding an effective selection marker that can be used in vivo for developing a stable transfection system of *B. microti*. 

## 4. Materials and methods

*B. microti* Peabody mjr strain (ATCC® PRA-99™) was intraperitoneally administered (10^8^ parasites) to BALB/c mice. Infected-RBCs (300–350 μL) were collected when parasitemia was between 25–30% and were cultured in vitro in T25 culture flask at 36.5 °C in an incubator with a humidified atmosphere (5% CO_2_ and 5% O_2_). The parasites were synchronized in an RPMI-1640 culture medium supplemented with L-glutamine (Gibco, Grand Island, NY, USA), 25 mM HEPES (Sigma, Tokyo, Japan), 0.85 g/L NaHCO_3_ (Wako, Osaka, Japan), and 20% fetal bovine serum (FBS) (Biowest, Courtaboeuf, France). Propidium iodide (Sigma) and Hoechst 33342 (Sigma) were used for confirming parasite viability by fluorescence microscopy (Keyence, Osaka, Japan).

Transfection was conducted using a single Nucleocuvette at a final volume of 100 μL, including 20 μg of circular plasmid construct in a 50 μL SF buffer and 50 μL of synchronized iRBCs (1.25–1.5 × 10^8^ parasites). The plasmid–iRBCs mixtures were transfected using program FA100 of the Amaxa 4D Nucleofection system (Lonza) and immediately transferred into 24-well culture plates with 1 mL of complete RPMI-1640 culture medium containing 5% fresh mouse RBCs. The luciferase activity was measured as described previously [8]. Briefly, the transfected iRBCs were ruptured by 10 times volume of 0.8% NH_4_Cl (Wako) at 24 h post-transfection, and the parasite pellet was lysed by luciferase assay substrate (Promega, Madison, WI, USA). Finally, the luminescence was measured using a 10 s integration interval by a GloMax®-Multi Detection System (Promega).

All the procedures were carried out according to ethical guidelines that were approved by Obihiro University of Agriculture and Veterinary Medicine (Permit for animal experiment: 19–120; DNA experiment: 1723-3; Pathogen: 201709-5). 

## Figures and Tables

**Table 1:** List of primers used in this study.

Element	Primer	Sequence (5′→3′)	Size (bp)	Reference
Promoter	*Bm 5′-actin* (*Hind* III-F)	GACGGTATCGATAAGCTTATCTTTGTTCCCTTTAGTAT	1252	This study
	*Bm 5′-actin* (*Hind* III-R)	GAATTCGATATCAAGCTTTTTATCTAAATTAGAATGTAATT		
	*Bm 5′-ef-1α-subunit* (*Hind* III-F)	GACGGTATCGATAAGCTTTCTTTTCTTTTGTGGCGA	1152	This study
	*Bm 5′-ef-1α-subunit* (*Hind* III-R)	GAATTCGATATCAAGCTTTTTTCTAACATTCAAGAGGCT		
	*Bm 5′-hsp70* (*Hind* III-F)	GACGGTATCGATAAGCTTTGTTATCATCAGTTACACGCAG	1314	This study
	*Bm 5′-hsp70* (*Hind* III-R)	GAATTCGATATCAAGCTTGTTGGCAGAAATTTCACTCC		
Reporter	Firefly luciferase (*Eco*R I-F)	AAGCTTGATATCGAATTCATGGAAGACGCCAAAAACAT	1653	Liu et al. [8]
	Firefly luciferase (*Eco*R I-R)	CCCGGGCTGCAGGAATTCTTACAATTTGGACTTTCCGCC		
Terminator	*Bb 3′-rap* (*Pst* I-F)	TTGTAAGAATTCCTGCAGGATGAGATGCGTTTATAATGGC	1281	Liu et al. [15]
	*Bb 3′-rap* (*Pst* I-R)	GGATCCCCCGGGCTGCAGCCTACGAACGATATGTCAAAGAG		

Restriction enzyme sites are underlined.
